# Clinical characteristics and prognosis of anti-alpha-Amino-3-Hydroxy-5-Methyl-4-Isoxazolepropionic acid receptor encephalitis

**DOI:** 10.1186/s12883-021-02520-1

**Published:** 2021-12-16

**Authors:** Zhe Zhang, Siyuan Fan, Haitao Ren, Lixin Zhou, Hongzhi Guan

**Affiliations:** Department of Neurology, Peking Union Medical College Hospital, Beijing and Peking Union Medical College and Chinese Academy of Medical Sciences, Beijing, 100730 China

**Keywords:** Antibody-mediated encephalitis, Autoimmune encephalitis, Alpha-amino-3-hydroxy-5-methyl-4-isoxazolepropionic acid receptor, AMPAR, Limbic encephalitis, Paraneoplastic encephalitis

## Abstract

**Background:**

Encephalitis associated with antibodies against alpha-amino-3-hydroxy-5-methyl-4-isoxazolepropionic acid receptor (AMPAR) is an extremely rare type of antibody-mediated encephalitis. This research aims to investigate the clinical characteristics and prognosis of anti-AMPAR encephalitis.

**Methods:**

This retrospective study enrolled nine patients with anti-AMPAR encephalitis. Demographic information, clinical manifestations, laboratory and radiological findings, treatment and response were collected and analyzed. These patients were followed up with an average period of 72 weeks to gather prognostic information.

**Results:**

Nine patients (7 females and 2 males) were enrolled with a mean age at disease onset of 59 years old. Three clinical pictures, including limbic encephalitis (*n* = 7; 78%), pure amnesia (*n* = 1; 11%) and fulminant encephalitis (*n* = 1; 11%) were identified. New symptoms of dysphagia and deafness were identified in the clinical spectrum of anti-AMPAR encephalitis. All patients had positive blood AMPAR antibodies, and six of them (67%) had paired positive antibodies in cerebrospinal fluid (CSF). Brain magnetic resonance imaging (MRI) was abnormal in 75% of the patients with no specific patterns recognized. Six patients (67%) had tumors, including lung cancers and thymomas. After immunotherapy and oncotherapy, partial improvement of neurological symptoms was observed among all 6 patients with available records during their hospitalization. After a mean follow-up of 72 weeks, 3 patients had marked decrease of modified Rankin Scale (mRS) score, 1 patient had unchanged mRS score, 4 patients died and the other one was lost.

**Conclusions:**

Anti-AMPAR encephalitis mainly presents as limbic encephalitis, and is paraneoplastic in 67% of cases. Thus, intensive screening for tumors is recommended for all anti-AMPAR patients. Although patients showed a good short-term therapeutic response, the overall prognosis was not satisfactory.

## Introduction

Antibody-mediated encephalitis constitutes a group of inflammatory brain diseases wherein antibodies target directly at cell-surface antigens of neurons and induce varieties of neuropsychiatric disturbances, including behavior changes, psychosis, amnesia, seizures, and altered consciousness state [1]. As the understandings of these diseases are deepened and the diagnostic tools mature, the incidence and prevalence of antibody-mediated encephalitis are increasing rapidly, even comparable to infectious encephalitis [2]. However, anti-AMPAR encephalitis is extremely rare. AMPARs are synaptic glutamate-gated cation channels composed of different combinations of four subunits, GluA1 to GluA4, widely expressed in the central nervous system. AMPARs mediate fast excitatory synaptic transmission crucial for ongoing fast information processing and synaptic plasticity, which are essential for diverse neurophysiological activities, such as learning and memory [3]. In 2009, Meizan Lai et al. first identified antibodies against GluA1/GluA2 of AMPARs in patients with limbic encephalitis, which was often paraneoplastic, responded well to immunological or oncological treatment, and tended to relapse [4]. Since then, less than 100 cases with diverse clinical manifestations have been reported in the literature, posing a great diagnostic challenge due to the clinical heterogeneity and rarity. By reviewing clinical profiles of the 9 patients diagnosed with anti-AMPAR encephalitis, we aim to summarize the clinical patterns and prognosis of the disease, thus promoting accurate diagnosis and prompt treatment.

## Material and methods

By retrospectively reviewing patients with positive AMPAR antibodies in CSF or serum, we identified 9 patients diagnosed with anti-AMPAR encephalitis between November 2014 and October 2019. Demographic information, clinical symptoms, laboratory studies (including CSF analysis, anti-neuronal antibodies in serum or CSF, scalp electroencephalogram (EEG), brain magnetic resonance imaging (MRI), and oncological screenings), treatment and response to treatment were retrieved from the patients’ medical records of outpatient visits and hospitalization. Specifically, clinical information of patient No.5, 6 and 9 was obtained by referring physicians and that of rest patients was collected by physicians in our center. Prognosis information was gathered during regular outpatient visits or telephone interviews. Both serum and CSF samples of all patients were tested for antibodies against neuronal cell surface antigens in our center. Written informed consents were obtained from every patient or next of kin/legally authorized representatives of the dead participant. This study was approved by the Research Ethics Committee of Peking Union Medical College Hospital.

### Screening for antineuronal antibodies

Anti-neuronal antibodies targeting both cell-surface and intracellular antigens, including N-methyl-D-aspartate receptor (NMDAR), contactin-associated protein-like 2 (CASPR2), AMPA1-R, AMPA2-R, leucine-rich glioma inactivated protein 1 (LGI1), γ-aminobutyric acid-B receptor (GABAB-R), glutamic acid decarboxylase 65-kilodalton isoform (GAD65), CV2/collapsin response mediator protein 5 (CRMP5), paraneoplastic Ma family (PNMA) 2, Ri, Hu, Yo, and amphiphysin, were tested by cell-based assays (CBA). All serum and cerebrospinal fluid (CSF) antibodies were measured using indirect immunofluorescence test kits purchased from EUROIMMUN AG (Lübeck, Germany) and used according to the manufacturer’s instructions. A dilution titer greater than or equal to 1:10 was considered positive.

## Results

### Patients and demographic information

We identified 9 patients with positive AMPAR antibodies in either serum or both CSF and serum from 2014 to 2019 in our center. Alternative diagnosis was reasonably excluded. Clinical information, including demographics, clinical presentations, laboratory findings, and comorbidities, was listed in detail in Table 1. Seven out of the 9 patients (78%) were female. The mean age at disease onset was 59 years with a range of 50–76 years. The mean time from symptom onset to diagnosis was 18 weeks (range 3–57 weeks). The average modified Rankin Scale (mRS) score during the initial visit was 4 (range 1–5) (Table 2).

**Table 1:** Clinical presentation of patients with anti-AMPAR encephalitis

Case Number	Age (years)/sex	Onset to Diagnosis (weeks)	Onset mode	Initial symptoms	Other symptoms presented during disease course	MRI (interval since disease onset)	EEG	CSF	AMPAR antibodies (sample type and titer)	Other antibodies	Tumor state
1	68/F	57	Subacute	Psychiatric disturbances	Confusion, amnesia, ataxia, dysarthria, urinary incontinence	Increased signal in right basal ganglia (10 days)	Diffuse low amplitude beta wave activity	Normal WBC, 55 mg/dL protein	CSF 1:32; blood 1:32	Blood Hu (+)	Not Found
2	52/M	32	Acute	Amnesia	Confusion, psychiatric disturbances	Increased signal in bilateral frontal subcortex (7 months)	NA	Normal WBC, 84 mg/dL protein	CSF (−); blood 1:10	(−)	Lung cancer by contrast-enhanced CT
3	51/F	20	Chronic	Amnesia	Sleep disorders, dizziness, right leg numbness and paresis	Increased signal in left medial frontoparietal lobe, right cingulate cortex, and bilateral cerebellar hemispheres (10 months)	NA	Normal WBC, 66 mg/dL protein	CSF 1:10; blood 1:32	(−)	Not Found
4	76/F	3	Acute	Psychiatric disturbances and amnesia	–	Normal (1 week)	Normal	WBC 15/μL, normal protein	CSF (−); blood 1:10	(−)	Small cell lung carcinoma by pathology
5	64/F	11	Chronic	Psychiatric disturbances and amnesia	Confusion	NA	NA	NA	CSF (−); blood 1:100	NA	Thymoma by pathology
6	50/F	NA	Subacute	Psychiatric disturbances and amnesia	Confusion, intermittent fever	Normal (2 weeks)	Normal	Normal WBC, normal protein	CSF (+); blood (+), both titers unknown	NA	Malignant thymoma (B3) by pathology
7	59/F	12	Subacute	Amnesia	Confusion, altered level of consciousness, psychiatric disturbances, involuntary movement, dizziness, right face and perioral numbness	Increased signal in left frontal lobe, left parietal lobe and right temporal lobe (2 months)	Diffuse abnormal (low amplitude and decreased slow wave activities)	Normal WBC, 58 mg/dL protein	CSF 1:100; blood 1:100	NA	Malignant thymoma (B3) by pathology
8	63/M	5	Chronic	Amnesia, psychiatric disturbances and ataxia	Confusion, sleep disorders, bilateral deafness, dysphagia	Increased signal in medial temporal lobes (9 days)	Slightly increased theta activity	Normal WBC, 86 mg/dL protein	CSF 1:10; blood 1:100	(−)	Not Found
9	51/F	3	Acute	psychiatric disturbances	Confusion, fever, apathy, dysarthria, dysphagia, arrythmia, difficulty in defecation and urination, central hypoventilation	Diffuse increased signal in bilateral cortex and subcortex (1 week)	NA	Normal WBC, normal protein	CSF (+); blood (+), both titers unknown	Blood Hu (+)	Possible thymoma by CT

*Abbreviations*: *CSF* Cerebrospinal fluid, *CT* Computed tomography, *EEG* Electroencephalogram, *MRI* Magnetic resonance imaging, *NA* Not available, *WBC* White blood cells

**Table 2:** Summarization of clinical profiles of patients with anti-AMPAR encephalitis

Demographics	Range	Mean	***N*** missing (%)
Sex		2 M/7F	0
Age (years)	50–76	59	0
mRS (initial)	1–5	4	0
mRS (last follow-up)	0–6	5	2 (22)
Onset to diagnosis (weeks)	3–57	18	1 (11)
**Clinical symptoms**	***N***	**positive**	***N*** **missing (%)**
Acute onset	3	33%	0
Subacute onset	3	33%	0
Chronic onset	3	33%	0
Amnesia	8	89%	0
Psychosis	8	89%	0
Ataxia	2	22%	0
Fever	2	22%	0
Sleep disorders	2	22%	0
Dysautonomia	2	22%	0
Numbness	2	22%	0
Dysarthria	2	22%	0
Dysphagia	2	22%	0
Deafness	1	11%	0
Altered levels of consciousness	1	11%	0
Involuntary movement	1	11%	0
Seizures	0	0%	0
**Laboratory and MRI findings**	***N***	**Positive**	***N*** **misssing (%)**
Only Blood AMPAR Ab (+)	3	33%	0
Only CSF AMPAR Ab (+)	0	0%	0
Blood and CSF AMPAR Ab (+)	6	67%	0
Other Onco-neuronal Abs	2	33%	3 (33)
Increased CSF protein	5	63%	1 (11)
Increased CSF WBC	1	13%	1 (11)
MRI abnormal	6	75%	1 (11)
EEG abnormal	3	60%	4 (44)
Tumor identified	6	67%	0

*Abbreviations*: *Ab* Antibody, *CSF* Cerebrospinal fluid, *CT* Computed tomography, *EEG* Electroencephalogram, *MRI* Magnetic resonance imaging, *mRS* Modified Rankin scale, *N* Number, *WBC* White blood cells

### Clinical presentation and MRI/EEG findings

Onset modes of the disease were acute in 3 patients, subacute in 3 patients, and chronic in 3 patients. The spectrum of disease presentation was broad with the most prominent symptoms as psychiatric disturbances (8 patients), confusion (8 patients), and amnesia (8 patients). We also identified symptoms such as fever (2 patients), paresthesia (2 patients), dysarthria (2 patients), dysphagia (2 patients), sleep disorders (2 patients), ataxia (2 patients), dysautonomia (2 patients), altered level of consciousness (1 patient), involuntary movement (1 patient), and deafness (1 patient) during the course of the disease. Among the 8 patients with available brain MRI, 6 (75%) had abnormal MRI findings, that is, T2/T2 fluid-attenuated inversion recovery (FLAIR) hyperintensities, which were not restricted to the limbic system, but also involved structures like cortex and subcortex, basal ganglia, and cerebellum.

Three major clinical pictures of anti-AMPAR encephalitis were identified according to disease onset modes and the prominent clinical symptoms. Seven patients manifested limbic encephalitis (LE), defined as the presence of at least two of the following symptoms: confusion, amnesia, and psychosis. One patient had pure amnesia and the remaining one had fulminant encephalitis.

The onset modes of patients with limbic encephalitis varied from acute (2 patients), subacute (3 patients) to chronic (2 patients). In addition to the typical LE symptoms, rare symptoms accompanied, such as ataxia, urinary incontinence, sleep disorders, dysphagia, dysarthria, dizziness, deafness, and involuntary movement. Interestingly, none of the patients showed typical limbic lesions in MRI. Two (patient No.4 and patient No.6) had normal MRI and the rest had increased T2/FLAIR signals in basal ganglia and frontal, temporal, and parietal lobes. The two patients with normal MRI also had normal EEG. EEG of patients No. 1, 7, 8 was non-specific decreased frequency or amplitude of brain waves, with no epileptic activity recorded.

**Fig. 1:**
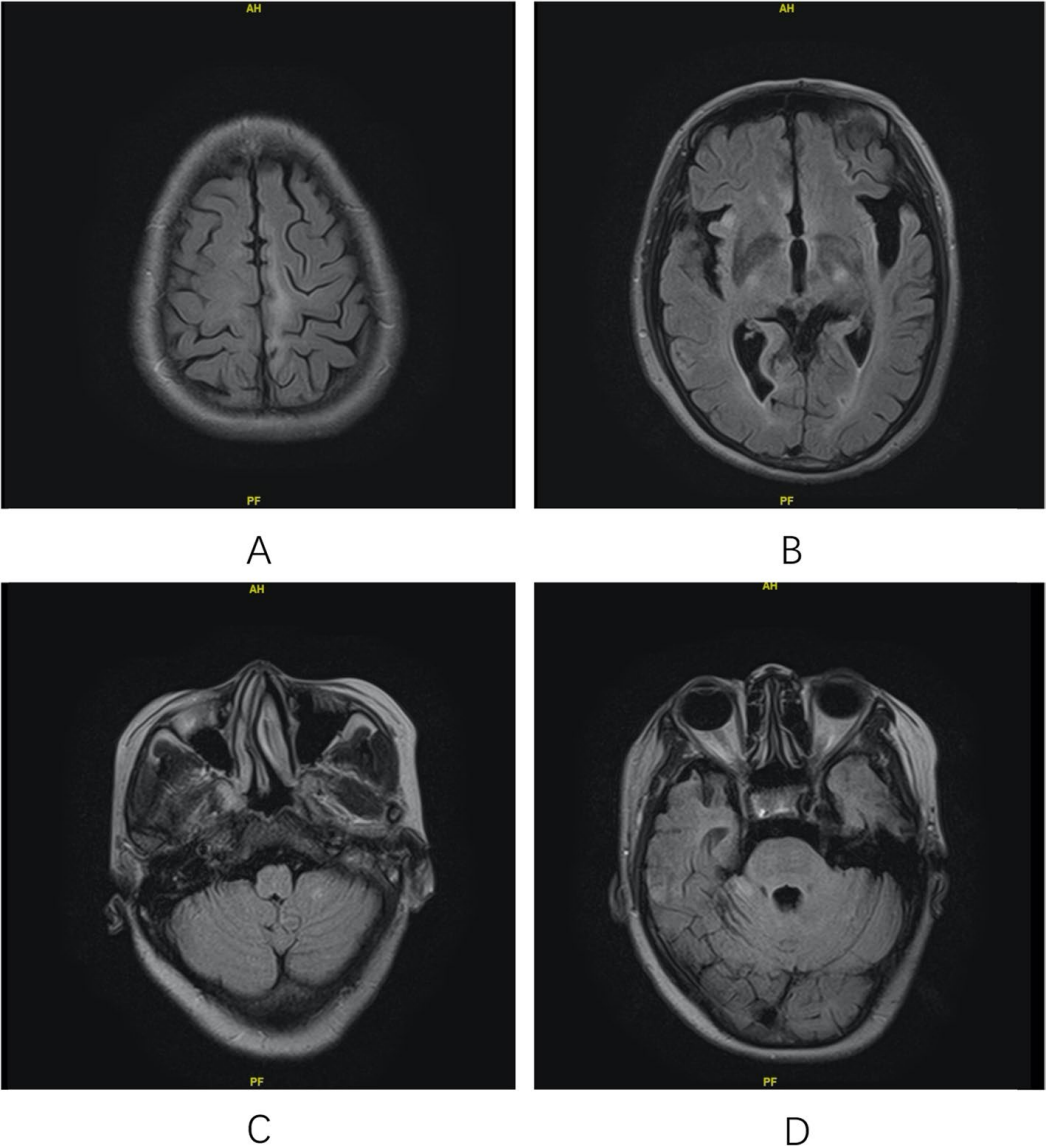
Brain MRI findings of patient No.3. The brain MRI was obtained 10 months after symptom onset, when the patient was admitted into hospital. Increased fluid-attenuated inversion recovery (FLAIR) signal could be observed in left medial frontoparietal lobe (**A**), right insular cortex (**B**), and bilateral cerebellar hemispheres (**C**, **D**). The signal abnormalities extended beyond the limbic system

For the patient with isolated amnesia as initial presentation (No.3), who was a 51-year-old female, the disease course was chronic. She gradually developed retrograde amnesia, insomnia, right leg numbness and paresis in 10 months. Electromyography indicated right-side neurogenic impairment at L5-S1 level. The brain MRI demonstrated increased signal in left medial frontoparietal lobe, right insular cortex, and bilateral cerebella hemispheres (Fig. 1), which extended beyond the limbic system and couldn’t fully explain her symptoms. The patient didn’t have psychosis and ataxia as anticipated.

Patient No. 9 was a 51-year-old female, who had a fulminant disease course and purely psychiatric symptoms without significant memory loss. Her symptoms were acutely onset during treatment of *Clonorchis Sinensis* infection, and within one week she quickly developed fever with a maximal temperature of 39.0 °C, as well as confusion, apathy, sialorrhea, dysarthria, difficulty in defecation and urination, arrythmia, and central hypoventilation. She was admitted into ICU and required mechanical ventilation. Her brain MRI showed diffuse bilateral abnormal signal in both cortex and subcortex areas, which was consistent with her symptoms.

### Laboratory findings

All patients except patient No.5 underwent lumbar puncture and the CSF samples were tested for routine and biochemical tests. 5 (63%) had elevated CSF protein ranging from 55 mg/dL to 88 mg/dL, while only 1 (13%) had elevated leukocytes of 15/μL.

All patients’ sera and CSF were tested for anti-neuronal antibodies as mentioned above. All patients had positive blood AMPAR antibodies, and six of them had paired positive antibodies in CSF. Antibody titers ranged from 1:10 to 1:100 in both sample types. None of the patients tested positive for AMPAR antibodies only in CSF samples. Although we tested antibodies for both GluA1 subunit and GluA2 subunit of AMPAR, only antibodies against GluA2 subunit were positive. In 6 patients who were tested for onco-neuronal antibodies, only patient No.1 and patient No.9 had positive Hu antibody in blood samples. Although Hu antibody was tumor-associated, only patient No.9 had thymoma, while no tumor was found in patient No.1 despite of intensive tumor screenings.

### Tumor state

Intensive tumor screenings including tumor marker panels, whole-body CT, PET-CT, and specific diagnostic tools for suspected tumors, such as mammography, and gastrointestinal endoscopy, were selectively applied in all nine patients. 6 (67%) patients were diagnosed with tumors, four of whom were pathologically confirmed with the rest two indicated by radiological findings. Two patients had lung cancers. One (patient No.3) had small cell lung cancer confirmed by tracheoscope-guided transbronchial lung biopsy and the other (patient No.9) had radiologically irregular soft-tissue mass in the anterior basal segment of the right lower lung lobe with multiple enlarged lymph nodes at right hilum and mediastinum, highly suggestive of malignant lung tumor. However, due to economic concerns, the patient and her families refused further assessment. Four patients had thymomas, three of which were confirmed with surgical pathology and one of which was radiologically suspected. In patient No. 6 and patient No. 7, the pathological types of thymoma were both B3, while that of patient No.5 was unavailable. Due to limited patient numbers, no obvious correlation between tumor state and demographic factors, clinical presentations, or prognosis was observed.

### Treatment and follow up

Six patients received first-line immunotherapy, including intravenous immunoglobulin (IVIG), steroids, and plasmapheresis (Table 3). The IVIG treatments were applied in standard dosage as 2.0 g/kg except for patient No.8, who had two rounds of standard IVIG treatment. Steroid treatments were administered in one to three pulses followed by maintenance dosage. Patient No.3 and patient No.8 received second-line immunomodulators, mycophenolate mofetil (MMF) and azathioprine (Aza), respectively. The rest three patients did not accept any immunotherapy. Patient No.2 received only symptomatic treatment for psychiatric disturbances and mood disorders. For the 6 patients with identified or suspected tumors, patient No.5 received surgical resection, and patient No.6 and No.7 received surgical resection and radiotherapy, according to the pathological type of tumors and the oncologists’ suggestions. The initial treatment response during hospitalization was positive for patients who received treatment in our hospital (patient No. 1–4, 7 and 8) despite of tumor state and therapy type, and that of the other patients was not available.

**Table 3:** Treatment and prognosis of patients with anti-AMPAR encephalitis

Case Number	Treatment	Short-term treatment response	mRS initial	mRS at last follow-up	follow-up (weeks)
1	IVIG, steroids	Significant improvement in cognition, psychosis and ataxia; urinary incontinence disappears	5	3	28
2	Symptomatic	Improvement in mood disorders and psychosis	3	6	23
3	IVIG, plasmapheresis, steroids, MMF	Numbness improved	4	2	75
4	IVIG, steroids	Improvement in amnesia	3	3	131
5	Tumor resection	NA	3	6	2
6	Tumor resection + radiotherapy	NA	NA	NA	NA
7	Tumor resection + radiotherapy, IVIG, steroids	Significant improvement in consciousness level and psychosis	5	0	214
8	IVIG, steroids, Aza	Improvement in psychosis	3	6	74
9	IVIG, steroids	NA	5	6	30

*Abbreviations*: *Aza* Azathioprine, *IVIG* Intravenous immunoglobulin, *MMF* Mycophenolate mofetil, *mRS* Modified Rankin scale, *NA* Not available

During the follow-up period ranging from 2 to 214 weeks, 3 patients (patient No. 1, 3, 7) had marked decrease of mRS score, one (patient No.4) had unchanged mRS score, 4 died (patient No.2, 5, 8, 9) and patient No.6 was lost, as shown in Table 3. Marked decrease of mRS score was defined as a decrease of at least 2 scores with an mRS score ≤ 3 at last follow-up. There was no significant survival difference between patients with and without tumors. Three out of the five patients with tumors died at last, among which patient No.2 and No.8 died of tumors, and patient No.9 died of subsequent multiple organ dysfunction syndrome comorbid with her fulminant encephalitis. One (No.8) of the three patients without tumors died of aspirational pneumonia.

No clinical relapse was observed in our patients. It’s noteworthy that patient No.7 with malignant thymoma and subsequent tumor therapy was later diagnosed as myasthenia gravis. After receiving regular immunotherapy, she remained asymptomatic.

## Discussion

In this article, we described 9 anti-AMPAR encephalitis patients and summarized their clinical characteristics. The patients were generally middle-aged women with an average onset age of 59 years old and a female-to-male ratio of 3.5: 1. Onset modes varied from acute, subacute to chronic. Three clinical pictures, including limbic encephalitis, pure amnesia and fulminant encephalitis, were identified with LE as the majority. Brain MRI were abnormal in 75% of the patients with no specific patterns recognized. All patients have positive blood AMPAR antibodies, and 67% of them have paired antibodies in CSF. 67% percent of the patients had tumors, lung cancers or thymomas. After immunotherapy and oncotherapy, partial improvement of symptoms was observed among all 6 patients during their hospitalization. During follow-up, 3 patients had marked decrease of mRS score, 1 patient had unchanged mRS score, 4 patients died and 1 was lost.

The demographic characteristics of anti-AMPAR encephalitis revealed by this research were similar to that of other studies [4–7]. In the original study that identified AMPAR as a novel antigen in 10 limbic encephalitis patients, the median age was 60 and 9 of the 10 patients were female. Additionally, in the recent systemic review covering 55 cases of anti-AMPAR encephalitis, the median age was 53.2 years old (range14–92 years) and the female-to-male ratio was 36 to 19 [4, 7].

Our research expanded the clinical features of Anti-AMPAR encephalitis. Anti-AMPAR encephalitis was initially recognized as limbic encephalitis, and the following research identified more clinical patterns [4]. Hoftberger et al. summarized four clinical modes in 22 patients, including limbic encephalitis (12 patients), diffuse encephalitis (8 patients), limbic encephalitis preceded by motor deficits (1 patient), and pure psychosis (1 patient) [5]. Similarly, Joubert et al. identified four main modes according to the prominent onset symptoms in a seven-patient cohort, including confusion (3 patients), isolated epileptic (1 patient), isolated amnestic (1 patient) and fulminant encephalitis (2 patients) [6]. Our study observed similar patterns, with limbic encephalitis in 7 patients, purely amnestic in 1 patient, and fulminant encephalitis in 1 patient. However, the clinical presentations were highly variable, ranging from the commonly seen symptoms of limbic encephalitis as psychosis, confusion, and amnesia, to the infrequent symptoms of seizure, dysautonomia, ataxia or other cerebellar symptoms, insomnia, involuntary movements, dysarthria, and sensory symptoms. We expanded the clinical spectrum of anti-AMPAR encephalitis by adding dysphagia and deafness. The bilateral deafness developed as a prominent symptom during the disease course without prior identifiable risks, such as ototoxic drugs administration. This manifestation was also observed in a recently diagnosed patient, which is not included in this series. The expanded profiles will help clinicians accurately recognize patients with atypical presentations, and reduce the rate of misdiagnosis and missed diagnosis.

Despite the diverse symptoms mentioned above in anti-AMPAR encephalitis, limbic encephalitis remains the majority. Additionally, clinicians should always meticulously rule out anti-AMPAR encephalitis in patients with pure amnesia or psychosis, as the disease is treatable and may be comorbid with tumors.

Limbic encephalitis is frequently seen in autoimmune encephalitis, such as anti-NMDAR, GABAB-R, CASPR2, LGI1, and AMPAR encephalitis, suggesting common mechanisms underlined. Limbic encephalitis in anti-AMPAR encephalitis is thought to be caused by antibody-mediated internalization of AMPAR clusters at synapses [4]. Increased availability of AMPAR clusters is critical for long-term potentiation in the hippocampus, and therefore for memory consolidation [8]. Specifically, GluA2 antibodies resulted in reduction of synaptic GluA2-containing AMPARs, impairment of long-term synaptic plasticity in vitro, and damaged learning and memory in vivo [9]. This explains amnesia in anti-AMPAR encephalitis patients and provides insights into the symptomatic overlap with LGI1 encephalitis, as LGI1-ADAM22 complex interacts with PSD95 and stabilizes AMPARs in the postsynaptic membrane [10, 11]. On the other hand, the glutamate hypothesis of psychosis indicates that hypofunction of GABAergic neurons may account for psychiatric symptoms in some autoimmune encephalitis. Indeed, internalization of NMDARs by GluN1 antibodies and AMPARs by GluA1/GluA2 antibodies affects the activities of cortical networks [3, 12]. Epilepsy is another commonly encountered symptom in autoimmune encephalitis. One possible mechanism is that increased seizure susceptibility is caused by reduced inhibitory neurotransmission, as indicated in GABAA-R, GABAB-R, or GAD65 encephalitis [13–16]. In hippocampal pyramidal neurons treated with CSF of anti-AMPAR encephalitis patients, patch-clamp revealed decreased miniature excitatory postsynaptic currents (EPSCs), which seemed paradoxical to seizures in patients [17]. Explanation was that decreased EPSCs resulted in decreased inhibitory synaptic transmission and increased intrinsic excitability, predisposing patients to epilepsy [17]. However, seizures were relatively rarely observed in anti-AMPAR encephalitis compared with other autoimmune encephalitis mentioned above. The discrepancy of seizure incidence and type in different antibody-mediated encephalitis remains unexplained.

Brain MRI, EEG, CSF study, and antibody test are the main diagnostic tools for anti-AMPAR encephalitis. Brain MRI is considered as a sensitive but not specific diagnostic tool for anti-AMPAR encephalitis. According to the systemic review with the largest anti-AMPAR encephalitis cohort (55 participants), up to 86% of the patients had abnormal brain MRI with a predilection of bilateral temporal lobes, which was related to topography of GluA1 and GluA2 expression [7]. Seventy-five percent of our patients had abnormal brain MRI, with no preference for specific brain sites. However, it should be noted that patients with anti-AMPAR encephalitis may have completely normal brain MRI as indicated by our patients and the imaging abnormalities may spread to unexpected sites, like basal ganglia, cerebellum, and even posterior temporal and parieto-occipital regions [18]. Therefore, for patients with nonspecific MRI manifestations but with typical symptoms, anti-AMPAR encephalitis should be cautiously differentiated. EEG was less sensitive than brain MRI and only 44% of patients had EEG abnormalities [7]. EEG was also nonspecific, varying from nonspecific slow waves, epileptiform activities, to normal. The most common EEG abnormality in our patients was nonspecific slowing. Cerebrospinal fluid study has limited significance for differential diagnosis. Systemic analysis revealed that inflammatory CSF changes, defined as pleocytosis, increased CSF protein levels, and/or oligoclonal band, were rather frequent seen in NMDAR, GABABR, AMPAR, and dipeptidyl-peptidase-like protein 6 (DPPX) encephalitis. While in autoimmune encephalitis with either CASPR2, LGI1, GABAA, or glycine receptor antibodies, CSF findings were generally normal [19]. In accordance with this systemic review, 5 patients (63%) in our study had elevated CSF protein and 1 (13%) patient showed pleocytosis in CSF. Blood and CSF AMPAR antibodies were the definitive diagnostic markers for anti-AMPAR encephalitis. Different from other studies, our study showed that the positivity rate of AMPAR antibodies was higher in serum than in CSF and that only GluA2 antibodies were detected. The difference might be accounted by the relatively small number of patients included. These findings suggest that for patients suspicious of anti-AMPAR encephalitis, both serum and CSF should be sent for antibody tests. Interestingly, the difference of clinical profiles between patients with antibodies against GluA1 and GluA2 was not clear yet.

Anti-AMPAR encephalitis can be paraneoplastic. Forty-eight to 70 % of patients were found to have tumors, mostly lung, thymus, breast, and ovarian tumors [4–7]. 6 (67%) patients in our study had tumors, 3 of which had lung cancers and the rest had thymomas. Additionally, rare tumors including medullary thyroid cancer, malignant melanoma, and Ewing’s Sarcoma were also reported in anti-AMPAR encephalitis cases [20–22]. Psychiatric symptoms at presentation predicted the presence of tumors [7]. These findings suggest the necessity of extensive tumor screening in patients with psychiatric symptoms. In addition to tumors, patients of anti-AMPAR encephalitis seem to have a predisposition to other autoimmune diseases. Systemic lupus erythematosus, Hashimoto’s thyroiditis, and myasthenia gravis were reported to be concurrent with anti-AMPAR encephalitis [23–25]. Therefore, signs of autoimmune diseases should also be paid attention to when anti-AMPAR encephalitis is suspected.

Treatment of anti-AMPAR encephalitis includes immunotherapy and oncological treatment if tumors are comorbid. Immunotherapy is composed of first-line therapies (IVIG, steroids, and plasmapheresis), and second-line therapies (rituximab and immunosuppressants, etc.). Treatment response, defined as mRS score decrease with an mRS score ≤ 3 at the last follow-up, was observed in 3 of 8 patients in our cohort and in 71% of the patients reported in the literature [5]. The overall survival rate of patients with and without tumors showed no significant difference as indicated by our study and literature [5]. The poor prognosis is unlikely related to delayed diagnosis and treatment, as indicated by the 3 patients who died despite of prompt diagnosis and adequate treatment. The presence of psychiatric symptoms and concurrent onco-neuronal antibodies were associated with poorer outcomes while younger age and confusion at presentation were linked with favorable prognosis [5, 7]. This was also observed in patients of anti-AMPAR encephalitis with concurrent CRMP5 antibodies [26]. Additionally, fulminant encephalitis was associated with a poor prognosis. Whether or not the presence of tumors or onco-neuronal antibodies predicts relapse remains elusive. Cases have been reported that anti-AMPAR encephalitis and the comorbid tumors relapsed after immunological and oncological treatments but not in our patients [27]. It seems that patients who received aggressive therapy (chemotherapy and rituximab) were unlikely to have relapses than those who did not [5]. Therefore, it’s important to closely monitor the patients after treatment.

Taken together, our study characterized a series of anti-AMPAR encephalitis patients from China and expand the clinical features of anti-AMPAR encephalitis. However, the limitations of the study are obvious. First, the small sample size limits the statistical power, therefore hindering a firm conclusion. Second, the clinical information was not documented completely, with the results of certain tests not available. Third, only patients clinically suspicious of encephalitis were tested for anti-neuronal antibodies. This selective bias may underestimate the unusual symptoms in anti-AMPAR encephalitis. Fourth, patients No.1 and patient No.9 were concurrent with positive blood anti-Hu antibodies, which complicated the diagnosis of anti-AMPAR encephalitis despite of paired positivity of anti-AMPAR antibodies in both serum and CSF. Fifth, for patients No. 2 and No. 4, despite of typical symptoms and the high positive predictive value of our method, low titers of anti-AMPAR antibodies were tested in only serum, raising the possibility of false-positive. Better understandings of this disease, including its symptoms, comorbidities, prognosis and development of better diagnostic and therapeutic maneuvers, rely on deeper investigations into the pathological mechanisms and the accumulation of patient cohorts.

## Conclusion

Encephalitis associated with antibodies against AMPAR is an extremely rare type of antibody-mediated encephalitis. Three clinical pictures, including limbic encephalitis, pure amnesia, and fulminant encephalitis were identified, with limbic encephalitis as the majority. Anti-AMPAR encephalitis is paraneoplastic in 67% cases and intensive screening for tumors is recommended for all anti-AMPAR patients. Although all patients showed a good short-term therapeutic response, the overall prognosis was not satisfactory.

## Data Availability

All data used and/or analyzed during the study is available on request from the corresponding author.

## Abbreviations

AMPAR: Alpha-amino-3-hydroxy-5-methyl-4-isoxazolepropionic acid receptor
Aza: Azathioprine
CASPR2: Contactin-associated protein-like 2
CBA: Cell-based assay
CRMP5: Collapsin response mediator protein 5
CSF: Cerebrospinal fluid
DPPX: Dipeptidyl-peptidase-like protein 6
EEG: Electroencephalogram
FLAIR: Fluid-attenuated inversion recovery
GABAB-R: γ-aminobutyric acid-B receptor
GAD65: Glutamic acid decarboxylase 65-kilodalton isoform
IVIG: Intravenous immunoglobulin
LE: Limbic encephalitis
LGI1: Leucine-rich glioma inactivated protein 1
MMF: Mycophenolate mofetil
MRI: Magnetic resonance imaging
mRS: Modified Rankin Scale
NMDAR: N-methyl-D-aspartate receptor
PNMA: Paraneoplastic Ma family
